# Neurosarcoïdose cérébrale

**DOI:** 10.11604/pamj.2018.30.67.15632

**Published:** 2018-05-28

**Authors:** Maha Ait Berri, Abdelhadi Rouimi

**Affiliations:** 1 Service de Neurologie, Hôpital Militaire Moulay Ismail, Meknès, Maroc

**Keywords:** Sarcoïdose, IRM, Neurologie, Sarcoidosis, MRI, neurology

## Abstract

We report the case of a 37-year old patient with right optic neuropathy. Magnetic resonance imaging (MRI) showed T2 hypersignal in the midline, enhanced after gadolinium injection (figure 1). Cerebrospinal fluid (CSF) analysis revealed lymphocytic meningitis with 64 white blood cells associated with hyperproteinorachy. Chest CT scan suggested the diagnosis of granulomatous inflammation, namely Stage 2 sarcoidosis. The level of serum angiotensin-converting enzyme (ACE) was high. The patient underwent corticosteroid therapy with good outcome. Sarcoidosis is a granulomatous diffuse, multisystemic disorder of unknown etiology. Neurologic sarcoidosis occurs in 5-15% of cases and neurologic symptoms suggest it in 10-30% of cases. Meningoencephalitis and cranial nerve involvement are the most common cliniconeurological manifestations, that are very varied. Facial nerve is the most common nerve to be affected, followed by the optic nerve. Brain MRI can better identify brain lesions, manifesting as infiltrating nodules in T1-weighted hyposignal and in T2-weighted hypersignal enhanced after contrast injection. It mainly affects the suprasellar region with involvement of the hypothalamus, the pituitary peduncle and the optic chiasm. Other anomalies are easily identified by gadolinium contrast agent, including diffuse or nodular thickening of the leptomeninges manifesting as pachymeningitis and lesions in the brain parenchyma (parietal, frontal, cerebellar regions) or in the spinal cord. The diagnosis is based on combination of clinical, radiological, laboratory tests and on histological data. Treatment is based on corticotherapy as first-line therapy sometimes associated with immunosuppressants.

## Image en médecine

**Figure 1:**
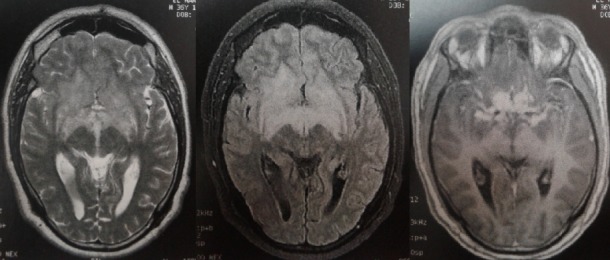
imagerie par résonance magnétique cérébrale montrant un processus expansif de la ligne médiane infiltrant hypersignal T2 FLAIR rehaussé après injection de gadolinium correspondant à une neurosarcoïdose

Nous rapportons l'observation d'un patient de 37 ans qui présenta une neuropathie optique droite. L'imagerie par résonnance magnétique (IRM) révéla la présence d'un hypersignal T2 de la ligne médiane rehaussé après injection de gadolinium (figure 1). L'examen du liquide céphalo-rachidien (LCR) retrouvait une méningite lymphocytaire à 64 éléments blancs avec hyperproteinorachie. Le scanner thoracique était en faveur d'une granulomatose inflammatoire type sarcoïdose stade 2. L'enzyme de conversion de l'angiotensine était élevée. Le patient a été mis sous traitement corticoïdes avec bonne évolution. La sarcoïdose est une granulomatose diffuse, multisystémique, d'étiologie inconnue. Une localisation neurologique est observée dans 5 à 15% des cas et révélatrice dans 10 à 30% des cas. Sa présentation clinique neurologique, très variée, est représentée essentiellement par la méningo-encéphalite et l'atteinte des nerfs crâniens. Le nerf facial est le plus fréquemment touché devant le nerf optique. L'IRM cérébrale permet de mieux visualiser les lésions sous forme de nodules infiltrant en isosignal T1 et en hypersignal T2, avec un rehaussement après injection de produit de contraste. La localisation préférentielle est suprasellaire avec atteinte de l'hypothalamus, du pédoncule hypophysaire et du chiasma optique. D'autres anomalies sont volontiers visualisées par le gadolinium, notamment un épaississement diffus ou nodulaire des leptoméninges avec un aspect de pachyméningite, des lésions du parenchyme cérébral (pariétal, frontal, cérébelleux) ou de la moelle épinière. Le diagnostic repose sur la combinaison de données cliniques, radiologiques, biologiques et histologiques. Le traitement repose sur la corticothérapie en première intention, en association parfois avec des immunosuppresseurs.

